# Sealing Ability of MTA and CEM Cement as Root-End Fillings of Human Teeth in Dry, Saliva or Blood-Contaminated Conditions

**Published:** 2010-11-15

**Authors:** Mohsen Hasheminia, Sam Loriaei Nejad, Saeed Asgary

**Affiliations:** 1. Department of Endodontics, Dental School, Isfahan University of Medical Sciences, Isfahan, Iran.; 2. General Practitioner, Tehran, Iran.; 3. Department of Endodontics, Iranian Center for Endodontic Research, Dental Research Center, Dental School, Shahid Beheshti University of Medical Sciences, Tehran, Iran.

**Keywords:** CEM Cement, Dental Leakage, Environment, MTA, NEC, Root-End Fillings

## Abstract

**INTRODUCTION:**

Sealing ability is an important factor for a root-end filling material in endodontic surgeries. This in vitro study aimed to compare the sealing ability of mineral trioxide aggregate (MTA) and a new endodontic cement named calcium enriched mixture (CEM) cement as root-end filling materials.

**MATERIALS AND METHODS:**

The experiments were carried out in dry, saliva or blood contaminated root-end cavities of hundred single-rooted extracted human teeth. After decoronation, the root canals were cleaned, shaped, obturated, and stored in 100% humidity for 5 days. Removing the apical 2-3mm of each root, a 3mm deep root-end cavity was ultrasonically prepared. Samples were randomly divided into 2 test groups of 45 roots/experimental material, and one subgroup (n=15) for each environmental condition as follows; a) dried before placing the filling material, b) filled after contamination with saliva, and c) filled after contamination with blood. Ten roots were used as positive/negative controls. Samples were placed in an incubator at 37°C for a day and immersed in methylene blue dye under reduced pressure environment for 48hours. Roots were sectioned longitudinally and examined under stereomicroscope by an independent observer.

**RESULTS:**

Using Kruskal-Wallis and Mann-Whitney U tests with Bonferroni correction, the results demonstrated significantly less leakage for the CEM cement in saliva contaminated condition when compared to MTA (P<0.001).

**CONCLUSION:**

It can be concluded that the sealing ability of CEM cement was superior to MTA in saliva contaminated condition.

## INTRODUCTION

The first treatment option in failed endodontics is retreatment of the teeth via an orthograde approach. When retreatment(s) have failed or cannot be accomplished by an orthograde approach, endodontic surgery is indicated [1]. The main objective of root-end fillings is to provide an apical seal that prevents the penetration of bacteria and their by-products into periradicular tissues from the root canal system. Therefore, the success of periradicular surgery is directly dependent on the achievement of a good apical seal, using a well adapted root-end filling material [2][3].

An ideal root-end filling material should be impervious to moisture, antibacterial, non-toxic, non-corrosive, non-resorbable, easy to manipulate, radiopaque, cost-effective, easily adaptable and adhesive to dentin. The ideal root-end filling should also be biocompatible with and promote regeneration of the periodontal apparatus [1][4].

Many different methods have been employed to assess endodontic microleakage. These include the use of bacteria, dye/ink, electrochemicals, use of radio isotopes and fluid filtration techniques [5][6]. The linear measurement of dye penetration is the easiest and most popularly employed method [6]. During last three decays, attempts have been made to correlate in vitro and in vivo studies with minimal success [7].

Various root-end filling materials have been suggested [4][8]. Among them, MTA is often used for comparison against other materials as it has shown promising results as a root-end filling material with a desirable feature of biocompatibility [9][10][11][12].

A new endodontic cement so called calcium enriched mixture (CEM) cement has been proposed that has been formulated using a different mixture of calcium compounds. Major components of CEM cement powder are CaO, SO_3_, P_2_O_5_, SiO_2_, and minor components are Al_2_O_3_, Na_2_O, MgO, and Cl as essential constituents [13], which provides a bioactive calcium and phosphate enriched material when being mixed with a water base solution, (compliant with the ISO 6876 standard for dental root canal sealing materials) [13][14]. Results of recent studies indicate that mixed CEM cement releases calcium and phosphate ions and then forms hydroxyapatite [15]. It has low cytotoxic effect on different cell lines, similar to MTA [16][17]. This material has also similar pH, increased flow, but decreased working time, film thickness, and estimated price than MTA [13].

The clinical uses of the CEM cement are similar to MTA. CEM cement has demonstrated similar results to MTA when used as pulp capping agent or furcation perforation repair [18][19]. It has also shown favorable results in pulpotomy of permanent molar teeth with established irreversible pulpitis and management of internal root resorption [20]. Furthermore, this material has an antibacterial effect comparable to calcium hydroxide and better than MTA or Portland cement [21]. Last but not least, CEM cement has shown lower mean dye leakage than commercial types of MTA and IRM in dry root-end preparations [3][22].

Researchers have reported that the sealing ability of MTA has not been compromized by blood or saliva contamination [23][24]. The purpose of this in vitro study was to evaluate the apical sealing ability of CEM cement compared to MTA in the various conditions (dry, saliva or blood-contaminated root-end cavities).

## MATERIALS AND METHODS

This study was approved by the Ethics Committees of Isfahan Medical University. One hundred freshly extracted, human single rooted teeth were used. The selection criteria dictated the presence of a single root canal and the absence of crack, fracture, root caries, or restorations. All teeth had mature apices and straight patent canals.

Crowns were removed at the CEJ. Working length was determined by subtraction of 0.5mm from the length from which a K-file (Mani, Japan) #15 was visible at the apical foramen. The apical enlargement of each root was carried out to a size #40 file and the remainder of the canal was flared using the step-back technique to a size #70 file. The canals were copiously irrigated with 5.25% sodium hypochlorite and finally rinsed with normal saline solution. The positive control was made up of five canals which were filled with gutta-percha alone and five canals were sealed entirely with sticky wax which was used as negative controls. The remaining root canals were obturated with laterally condensed gutta-percha (DiaDent, Korea) and AH26 sealer (Dentsply, Maillefer, Tulsa, USA). After filling the access cavities with Coltosol (Coltene, Altstatten, Switzerland) the teeth were placed in a 37°C and 100% humidity incubator for five days. Then root-end resections were made by removing 3mm from the root-end at a 90 degree angle to the long axis of the root with a diamond disc (D&Z, Darmstadt, Germany). A 3mm deep root-end cavity was prepared ultrasonically, powered by a minipiezon with DT-043 ultrasonic retrotip (EMS, Nyon, Switzerland). The teeth were then coated with two layers of nail varnish and one layer of sticky wax except for the resected root-end surface. The prepared roots were randomly divided into following groups and subgroups:

1) Forty-five filled root canals were divided into three subgroups of 15 canals each. ProRoot MTA, (tooth colored formula, Dentsply, Tulsa, OK, USA) was mixed according to the manufacturer’s instructions and packed into the cavities in the following conditions: 1a) dry (the root-end cavities were dried prior to filling); 1b) blood (the root-end cavities were contaminated with human blood prior to filling) and 1c) saliva (the root-end cavities were contaminated with human saliva prior to filling); and 2) Forty-five filled root canals were divided into three subgroups of 15 canals each. The CEM cement was mixed according to the developer’s instructions and placed in the prepared root-end cavities with the following conditions: dry (subgroup 2a), blood-contaminated (subgroup 2b) and saliva-contaminated (subgroup 2c).

Samples were incubated for 24 hours at 37°C and 100% humidity. The roots were then immersed in 2% (0.02g/1mL) methylene blue dye at reduced pressure for 48 hours. Afterwards the roots were rinsed in running tap water for 10 minutes. They were then grooved on the buccal and lingual surfaces and split longitudinally into two sections. Linear dye penetration was measured in millimeter using a stereomicroscope (SZX9/12, Olympus, Tokyo, Japan) with a 0.1mm ocular grid (U-OCMSQ10/10, Eyepiece Micrometer, Olympus) at×10 magnification. The results were analyzed by Kruskal-Wallis and Mann-Whitney U tests. Statistically significant differences among the groups were set at P<0.05.

## RESULTS

**Figure 1:**
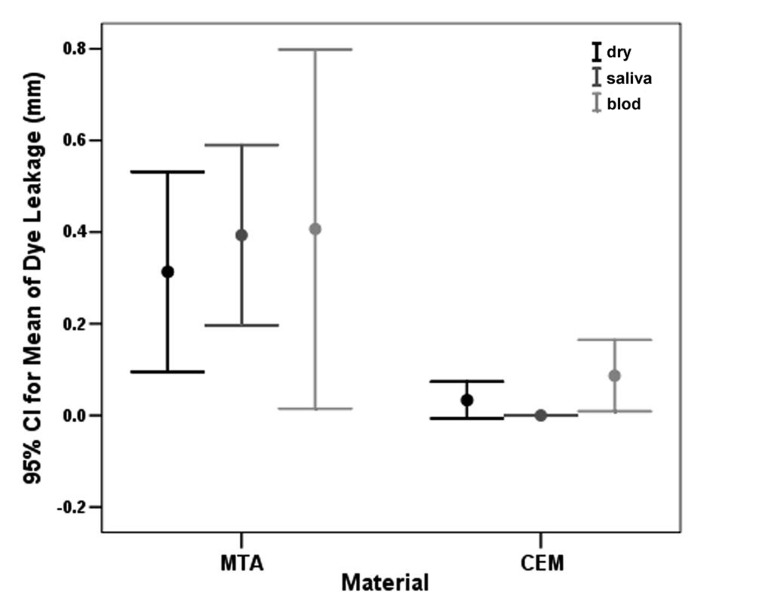
Bar chart showing the mean dye penetration values in the experimental groups and different environments at a 95% confidence level

The positive control specimens all displayed total dye penetration, while the negative controls all showed no evidence of dye penetration. All experimental subgroups demonstrated dye penetration except for group 2c (CEM cement in saliva). MTA samples sealed best in a dry environment though their seal was still not as successful as CEM cement test groups (Figure 1, Table 1).

**Table 1:** Statistical indices of linear dye penetration of test materials in different environments

**Materials**	**Statistical indexes**	**Dry (n=15)**	**Saliva (n=15)**	**Blood (n=15)**
**MTA** **(n=45)**	**Mean (SD)**	0.31 (0.39)	0.39 (0.35)	0.40 (0.70)
	**Median**	0.1	0.3	0.1
	**Mean Rank**	55.93	62.70	55.70
	**Range**	1.3-0.0	1.1-0.0	2.7-0.0
**CEM** **(n=45)**	**Mean (SD)**	0.03 (0.07)	0.0 (0.0)	0.08 (0.14)
	**Median**	0.0	0.0	0.0
	**Mean Rank**	32.63	25.50	40.53
	**Range**	0.2-0.0	0.0-0.0	0.4-0.0

Mann-Whitney U analysis revealed that dye penetration values between the two tested materials (MTA and CEM), regardless to environments, was significant (P<0.001).

Kruskal-Wallis test did not show any significance difference among three used environments, regardless to test materials (P=0.988).

To assess interaction between materials (n=2) and environments (n=3) Kruskal-Wallis test was used which revealed statistical significance (P<0.001). Mann-Whitney U tests with Bonferroni correction for pair comparisons (n=15) were performed. While there were a more effective seal in the saliva contaminated CEM subgroup than MTA subgroup (P<0.001), their seal was similar in dry and blood-contaminated environments (P=0.007 and P=0.079, respectively). Pair comparisons among different environments for each material revealed no significant differences (P=0.007).

## DISCUSSION

Apical seal during periradicular surgery may be compromised by contamination of the root-end cavity which is sometimes inevitable. This in vitro experiment was therefore designed to test the seal of two root-end fillings in dry, blood or saliva contaminated conditions, so as to simulate often found clinical conditions.

Several methods have been employed to assess apical microleakage [5][6][25]. There is a lack of evidence to suggest that the use of any particular method is superior to another. Dye penetration methods are commonly used for microleakage studies [6] because dyes are cheap, safe, readily available, relatively easily stored and used, and their penetration can be evaluated quantitatively [26]. Entrapped air may influence the results however this problem may be solved by using a reduced pressure treatment as performed here [27].

Some researchers have suggested that dye particles such as methylene blue are considerably smaller than microorganisms and their by-products [9]. This may lead to greater in vitro microleakage than may be expected clinically [28]. However, if smaller ions or molecules cannot permeate a restorative material then leakage of bacteria will be also prevented [6][9]. Therefore a more stringent test with a smaller tracer is still useful clinically if a seal is demonstrated. In this study, linear measurement of dye penetration under reduced pressure was carried out to allow discrimination of the sealing ability of the two materials. The clinical relevance remains uncertain.

A recent in vitro study showed that the long term sealing ability of MTA was not the best [29], however, methylene blue dye penetration studies have demonstrated seal of MTA, superior to other popular root-end fillings [3][7][30][31]. except for CEM cement [3][22]. The results of the present study showed a better sealing ability for CEM cement even when contaminated with blood or saliva.

Torabinejad et al. also assessed root-end dye microleakage in dry and blood contaminated environments and reported that MTA sealed better than amalgam, IRM (L.D. Caulk) and Super EBA (Bosworth Co., Skokie, IL) [23]. They found that the mean dye leakage of MTA contaminated with blood was similar to that in a dry environment, concurring with our results.

In this study linear dye penetration was not affected by environmental factors. Farhad et al. demonstrated that the difference in linear dye penetration in root-ends filled with MTA in dry, blood contaminated and saliva contaminated environments was not statistically significant, which concurs with the results of the present study [24]. Lower mean dye penetrations using CEM cement specimens instead of MTA under dry conditions have also been reported, concurring with the present study [3][22].

The excellent seal of CEM cement, particularly in saliva contaminated environment, was thought to be due to several physical and chemical characteristics of this novel material. CEM cement provides good handling characteristics; once mixed, this cement does not adhere to the applicator and is easily adoptable [3]. It is a water-base cement; in this regards, the moisture not only adversely affects this material, but also influences the chemical reactions which lead to more hermetic seal and hardening process. Saliva increased the wetting of the dentinal walls, enables adaptation of CEM cement within irregularities of root canal walls, and also facilitates its penetration into the dentinal tubules [32]. Slight expansion of CEM cement after being used as root-end filling material (0.075mm according to the International Organization for Standardization ISO 6876-2001: Dental root sealing materials) is in appreciable contrast with other root-end fillings which dominantly present microleakage due to setting shrinkage [12]. This property of CEM cement leads to much better adaptation of this material to the root-end cavity walls. High percentage of small particles (0.5-2.5µm) in this material supports this cement’s access to dentinal tubules with inner diameter range of 2-5µm [32]. Furthermore, in the presence of an aqueous environment, this biomaterial produces a large amount of hydroxyl, calcium, and phosphate ions which readily raise the local pH [15], hydroxyapatite formation [33] and antibacterial activity [16]. Hydroxyapatite formation provides an additional seal at the interface of the material and cavity walls, and also the surface of the filling area [14][32][33]. All the above explains CEM cement ability to prevent or significantly reduce microleakage. These properties may lead to better wetting, penetrance, filling, and sealing, and easier manipulation in the moist oral environment.

## CONCLUSION

Within the limitations of this in vitro study, the results indicate that CEM cement is a more effective root-end sealant than MTA and is largely unaffected by salivary contamination. This is particularly important when this material is used for root-end filling where the blood and periradicular fluids are often difficult to control. However, further ex vivo and in vivo studies are needed to assess additional properties of this novel material.
